# Impact of varying air cavity on planning dosimetry for rectum patients treated on a 1.5 T hybrid MR‐linac system

**DOI:** 10.1002/acm2.12903

**Published:** 2020-05-23

**Authors:** Paola Godoy Scripes, Ergys Subashi, Sarah Burleson, Jiayi Liang, Paul Romesser, Christopher Crane, James Mechalakos, Margie Hunt, Neelam Tyagi

**Affiliations:** ^1^ Department of Medical Physics Memorial Sloan‐Kettering Cancer Center New York NY USA; ^2^ Department of Radiation Oncology Memorial Sloan‐Kettering Cancer Center New York NY USA

**Keywords:** 1.5 T magnetic field, electron return effect (ERE), MR‐guided radiation therapy, rectal cancer

## Abstract

**Purpose:**

To investigate the dosimetric impact of magnetic (B) field on varying air cavities in rectum patients treated on the hybrid 1.5 T MR‐linac.

**Methods:**

Artificial air cavities of varying diameters (0.0, 1.0, 1.5, 2.0, 2.5, 3.0, and 5.0 cm) were created for four rectum patients (two prone and two supine). A total of 56 plans using a 7 MV flattening filter‐free beam were generated with and without B‐field. Reference intensity‐modulated radiation therapy treatment plans without air cavity in the presence and absence of B‐field were generated to a total dose of 45/50 Gy. The reference plans were copied and recalculated for the varying air cavities. D_95_(PTV_45_–PTV_50_), D_95_(PTV_50_–aircavity), V_50_(PTV_50_–aircavity), D_max_(PTV_50_–aircavity), and V_110%_(PTV_50_–aircavity) were extracted for each patient. Annulus rings of 1‐mm‐diameter step size were generated for one of the air cavity plans (3.0 cm) for all four patients to determine D_max_ (%) and V_110%_ (cc) within each annulus.

**Results:**

In the presence of B‐field, hot spots at the cavity interface start to become visible at ~1 cm air cavity in both supine and prone positioning due to electron return effect (ERE). In the presence of B‐field D_max_ and V_110%_ varied from 5523 ± 49 cGy and 0.09 ± 0.16 cc for 0 cm air cavity size to 6050 ± 109 cGy and 11.6 ± 6.7 cc for 5 cm air cavity size. The hot spots were located within 3 mm inside the rectal‐air interface, where D_max_ increased from 110.4 ± 0.5% without B‐field to 119.2 ± 0.8 % with B‐field.

**Conclusions:**

Air cavities inside rectum affects rectum plan dosimetry due ERE. Location and magnitude of hot spots are dependent on the size of the air cavity.

## 1 INTRODUCTION

Magnetic resonance (MR)‐guided radiation therapy using a hybrid MR‐linear accelerator (linac) system has become clinically available using commercial systems in recent years.^1^, ^2^ These systems have enabled methods of increased treatment precision under real‐time guidance and online plan adaptation. One such system is the Unity 1.5 Tesla (T) hybrid MR‐linac system with a 7 MV Elekta linac and a high‐field strength 1.5 T Philips MR magnet.^3^ Higher‐field strength hybrid systems provide superior MR image quality for daily adaptation and therapy response assessment, but pose multiple dosimetric challenges including electron return effect. The electrons in the presence of a magnetic field scatter and their trajectories are influenced by the resultant Lorentz force. When the electrons move from a high‐density medium to a low‐density medium, the electrons loop back due to Lorentz force and re‐enter the high‐density medium. This results in a dose enhancement at the interface of the high and low media, often called the electron return effect (ERE).^4^, ^5^, ^6^, ^7^ In the presence of 1.5 T magnetic field, the radius of curvature of the electrons looping back can be as much as 1 cm. Various methods have been suggested to compensate for ERE such as the use of opposed beams or including the impact of magnetic fields in the inverse planning optimization which works well for stationary air cavities but not for variable air volume.^8^ Electron return effect can pose a dosimetric concern for rectum patients treated on the hybrid MR‐linac system using intensity‐modulated radiation therapy (IMRT) as the air in the rectum is not consistent from day‐to‐day and may even change during the daily online plan adaptation process that can take as long as 45–60 min.^9^ The goal of this study is to investigate and predict the effect of the magnetic field on rectum planning dosimetry due to the electron return effect. We specifically sought to investigate the impact and location of ERE on plan coverage and plan hot spots for rectum patients with air cavities of varying size, treated in both supine and prone positions.

## 2 MATERIALS AND METHODS

**FIG. 1:**
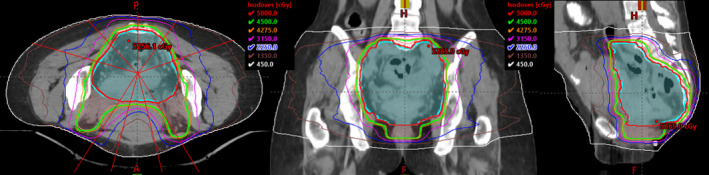
Typical dose distribution for a two‐level PTV (PTV45/PTV50) rectum plan using IMRT and simultaneous integrated boost (SIB) techniques.

Four patients previously treated at our institution with IMRT on a conventional linac were selected for this study. Patients were treated either in prone position on a belly board (mid and upper rectal) or supine (distal rectal) position in a customized aquaplast immobilization mold. All four of these patients received a total dose of 45 Gy to at‐risk lymph nodes and 50 Gy to gross disease in 25 fractions using IMRT and simultaneous integrated boost (SIB) techniques. Figure 1 shows a typical beam arrangement and IMRT dose distribution, for example, rectum patient treated on a conventional linac. Our department constraints for 25 fractions dose regime are listed in Table 1.

**TABLE 1:** Department constraints for pelvis/rectum 25 fractions regime. PTV45DVH excludes PTV50 from PTV45.

Structure	Dosimetric criterion
PTV50	Dmax < 5500 cGy
	V5000 cGy> 90%
	D95%> 5000 cGy
PTV45DVH	V4500 cGy> 90%
	D95%> 4500 cGy
Small bowel	Dmax < 5500 cGy
	V4500 cGy < 40 cc
Large bowel	Dmax < 6500 cGy
Bladder	V6600 cGy < 30%
External genitalia	V3000 cGy < 20%
	V2000 cGy < 67%
Cauda	Dmax < 6500 cGy
Femoral heads	V4500 cGy < 20%

**FIG. 2:**
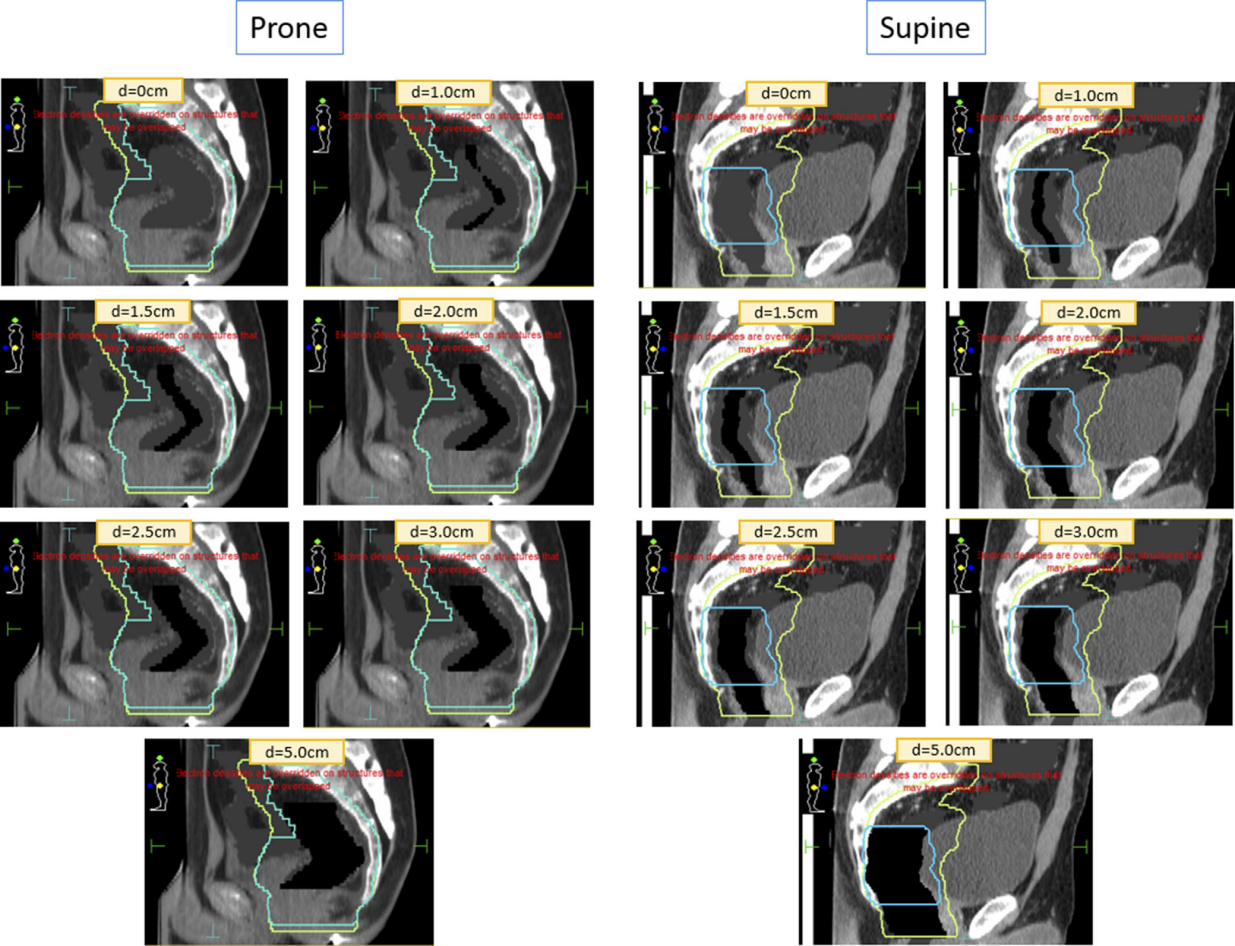
Air cavities of varying diameter (0, 1.0, 1.5, 2.0, 2.5, 3.0, and 5.0 cm) for prone and supine rectum patients. Cyan: PTV 50; Green: PTV 45.

To evaluate the effect of magnetic field on changing air volumes in rectum patients, two prone and two supine patients were selected, and artificial air cavities of diameters 0.0, 1.0, 1.5, 2.0, 2.5, 3.0, and 5.0 cm were created for all four patients. Air cavities extended superiorly and inferiorly within the high‐dose PTV volume following the length of rectum anatomy, yielding a “tube”‐like structure as shown in Fig. 2. For each patient, a reference plan with no air cavity and in the presence of magnetic field was optimized following department guidelines for target coverage/OARs constraints and prescribed to a total dose of 45 Gy to nodes and 50 Gy to gross disease in 25 fractions using IMRT and simultaneous integrated boost planning. Plans were generated using a 7 MV flattening filter‐free beams and step and shoot delivery with the following settings: minimum segment area of 4 cm^2^, minimum segment width of 0.5 cm, minimum MU/seg of 5 MUs, and a total of 100 segments per plan. Nine beams spread around the patient, as shown in Fig. 1, avoiding entrance through high‐density couch material were used. Plans were calculated in Monaco version 5.4.0 treatment planning system using Unity beam data and the GPU Monte Carlo calculation algorithm (GPUMCD) with 0.3 cm grid size and 1% statistical uncertainty per calculation.^10^, ^11^ For patients with air gas inside bowel/rectum on the initial planning CT scan, both rectum/bowel contours were assigned density of water (relative ED = 1) during planning due to the fact that the air cavity will not be consistent from day to day. For one case where small and scattered air pockets (few voxels in volume) were present, no density override was done on initial planning/optimization since its impact on dose calculation was assumed minimal.

To evaluate the effect of varying air cavity during online adaptive workflow in the presence of magnetic field, the initial reference plan was copied and recalculated (using same segments and MUs) for each new artificial air cavity diameter scenario with its relative electron density assigned to 0.18 (−824 HU), as shown in Fig. 2. Consequently, a total of seven plans in the presence of magnetic field were created for each patient: reference plan with no air cavity, 1.0‐, 1.5‐, 2.0‐, 2.5‐, 3.0‐, and 5.0‐cm‐diameter air cavity plan. To investigate the effect of the ERE alone due to magnetic field, those seven scenarios were also evaluated without the presence of magnetic field: a different reference plan with no air cavity and with the same IMRT constraints was generated using the Unity research beam model with magnetic field turned off, and then copied and recalculated (using same segments and MUs) for the different air cavity diameters. A total of 56 plans were generated for this study.

### 2.A Plan analysis

**FIG. 3:**
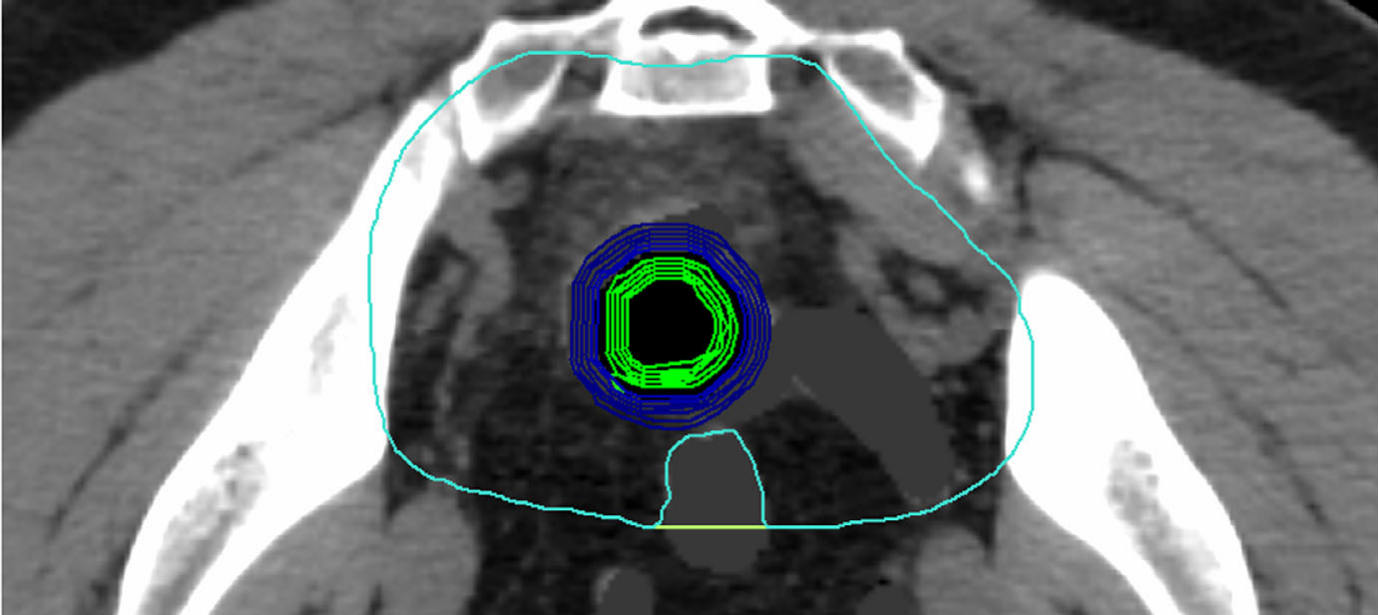
One mm annulus rings generated inside PTV 50 to determine the hot spots volume and maximum dose due to ERE. Green annulus rings represent rings generated inwards from the rectum surface. Blue annulus rings represent rings generated outwards from the rectum surface.

The following dosimetric parameters were extracted for each patient: D_95_ PTV45DVH (PTV45 excluding PTV50), D_95_(PTV50‐aircavity), V_50_(PTV50‐aircavity), D_max_(PTV50‐aircavity), and V_110%_(PTV50‐aircavity). Box plots with mean and standard deviation (along with individual data points) were generated for each of the above dosimetric parameters and plotted against air cavity diameter size. A polynomial fit was also performed to predict the magnitude of dose hot spots as a function of air cavity diameter size. To determine the location of hot spots due to ERE, annulus rings of 1‐mm‐diameter step size were generated for one of the air cavity plans (3.0 cm diameter) for all four patients. Five annulus rings inwards and outwards from the cavity interface were generated as shown in Figure 3. Hot spots maximum dose and volume [D_max_ (%), V110% (in cc)] within each annulus was extracted for analysis. To obtain realistic values within the annulus, the dose calculation for 3 cm air cavity plan was redone using a 1 mm grid size.

## 3 RESULTS

**FIG. 4:**
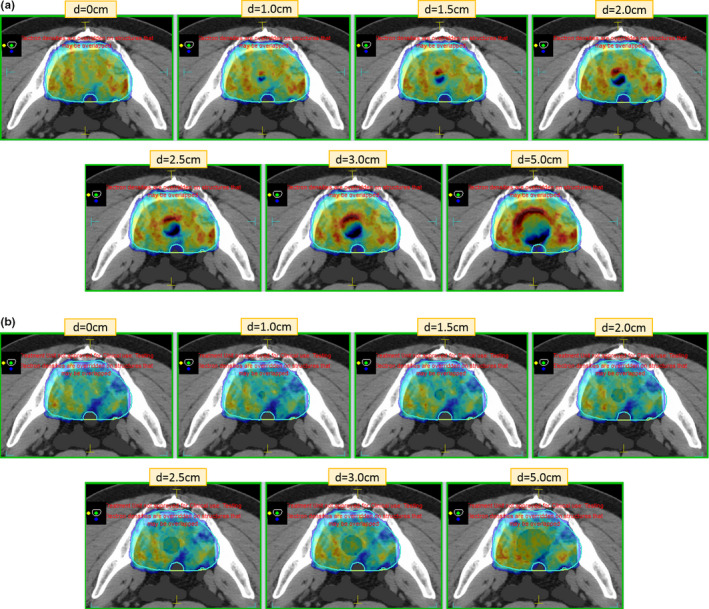
Dose color wash in axial plane, for example, patient in prone positioning (a) in the presence of B field and (b) in the absence of B field. Dose color wash isodose settings: minimum 45 Gy (dark blue) and maximum 56 Gy (red).

**FIG. 5:**
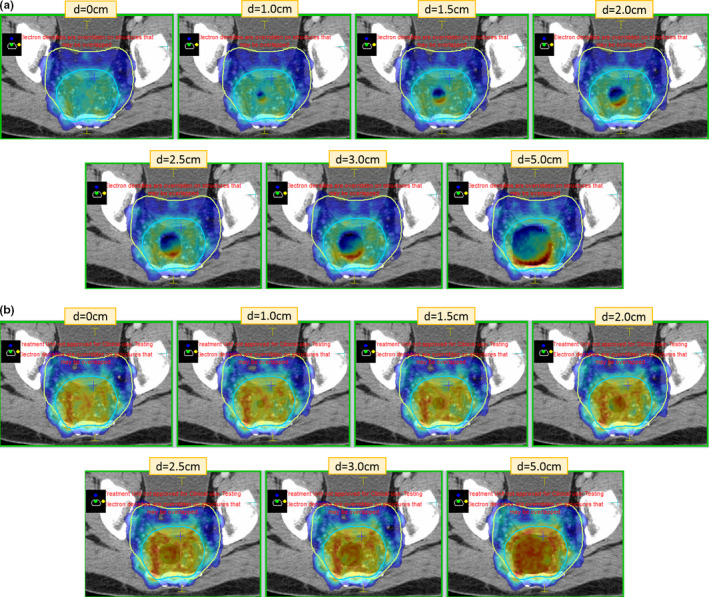
Dose color wash in axial plane, for example, patient in supine positioning (a) in the presence of B field and (b) in the absence of B field. Dose color wash isodose settings: minimum 45 Gy (dark blue) and maximum 56 Gy (red).

Figures 4(a) and 4(b) shows the dose color wash on an axial slice, for example, prone patient with and without the B field. Figures 5(a) and 5(b) shows similar dose color wash, for example, patient in supine position. For both prone and supine positions, five beams posteriorly and four beams anteriorly were planned, similar to a conventional linac plan done in our department, as shown in Fig. 1. In the presence of magnetic field, hot spots with opposing cold spots at the cavity interface due to ERE start to become visible at approximately 1 cm air cavity in both supine and prone positioning. The volume and magnitude of these hot spots also increases with increasing air cavity size. No such localized hot spots are visible on plans in the absence of magnetic field with increasing air cavity size in both prone and supine position. On the contrary, hot spots are spread within the volume and cold spots are barely visible and are within the Monte Carlo statistical uncertainty.

### 3.A PTV coverage vs air cavity size

The effect of air cavity size on PTV coverage was evaluated using D_95_ on the PTV45DVH (which excludes PTV50 from PTV45) volume as well as D_95_ and V_50_ on PTV50 minus Air. Change in PTV45DVH D_95_ was minimal for both the presence and absence of B field. In the presence of the 1.5 T magnetic field, D_95_ varied from 4459 ± 49 cGy for 0 cm air cavity size to 4463 ± 44 cGy for 5 cm air cavity size and, in the absence, varied from 4470 ± 42 cGy for 0 cm air cavity size to 4486 ± 47 cGy for 5 cm air cavity size.

**FIG. 6:**
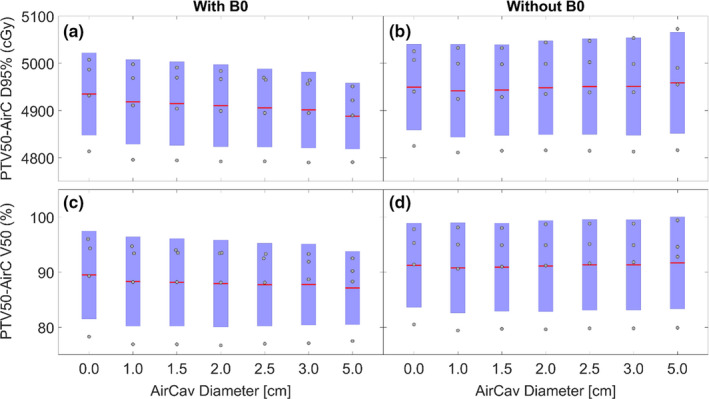
D_95_ and V_50_ coverage of (PTV50–air cavity volume) structure in the presence (a, c) and absence (b,d) of magnetic field.

The effect of air cavity size on the high‐dose volume (PTV50) was higher. Because of varying air volume inside high‐dose PTV and considering the dosimetry inside air cavity to be clinically irrelevant, the effect was evaluated using D_95_ and V_50_ on (PTV50 minus air) volume as shown in Figs. 6(a)–6(d). Change in D_95_ with air cavity size in the absence of B field was minimal (<10 cGy dose difference). In the presence of the B field, D_95_ for PTV50 minus air varied from 4935 ± 87 cGy for 0 cm air cavity size to 4882 ± 69 cGy for 5 cm air cavity size (<1% difference). There was 2.4% drop in V50 with varying air cavity in the presence of magnetic field (89.5 ± 8.0% for 0 cm air cavity size to 87.1 ± 6.7 % for 5 cm air cavity size).

### 3.B PTV hot spot versus air cavity size

**FIG. 7:**
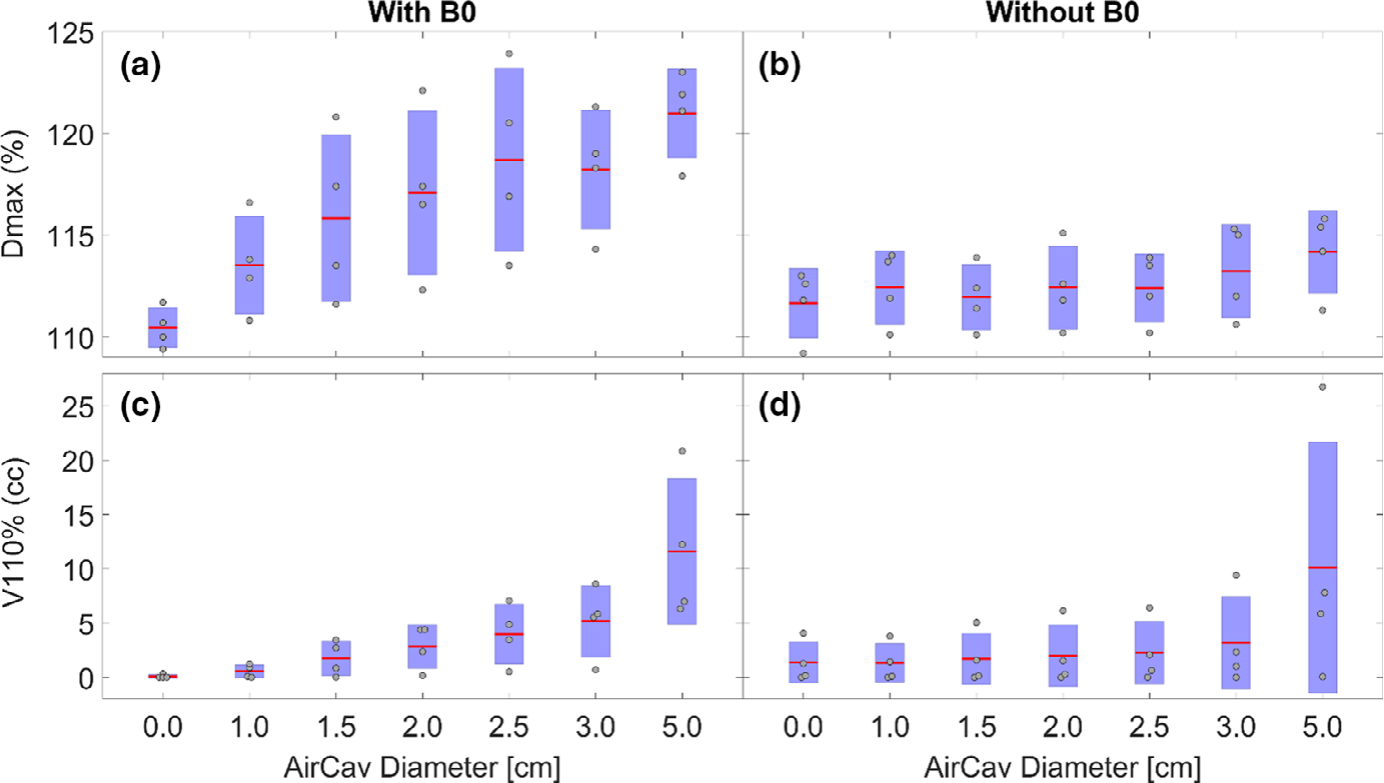
D_max_ and V_110%_ coverage of (PTV50–air cavity volume) structure in the presence (a,c) and absence (b,d) of magnetic field.

**FIG. 8:**
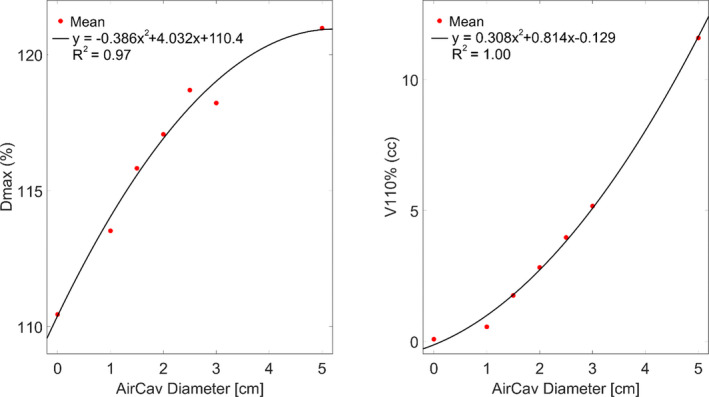
D_max_ and V_110%_ polynomial fit for (PTV50–air cavity volume) structure in the presence of magnetic field.

PTV50‐ air cavity D_max_ and V_110%_ experienced the greatest dosimetric impact due to varying air cavity size as shown in Figs. 7(a)–7(d). In the presence of magnetic field, D_max_ varied from 5523 ± 49 cGy for 0 cm air cavity size to 6050 ± 109 cGy for 5cm air cavity size. The corresponding change in hot spot volume, described as V_110_ (in cc) was 0.09 ± 0.16 cc for 0 cm air cavity to 11.6 ± 6.7 cc for a 5 cm air cavity size. A polynomial fit to the D_max_ (%) and V_110%_ (cc) values was performed to predict hot spot values with varying air cavity size (Fig. 8). The predicted hot spot values (in % as well as cc) are calculated as:

$$\mathrm{PTV}\;D_{\max}\left(\%\right)=-0.386{\left(\mathrm{Aircavity}\;\mathrm{diameter}\right)}^{2}+4.302\left(\mathrm{Aircavity}\;\mathrm{diameter}\right)+110.$$

$$\mathrm{PTV}\;V_{110\%}\left(\mathrm{cc}\right)=0.308{\left(\mathrm{Aircavity}\;\mathrm{diameter}\right)}^{2}+0.814\left(\mathrm{Aircavity}\;\mathrm{diameter}\right)-0.129$$

### 3.C Location and volume of ERE hot spots: Annulus results

**FIG. 9:**
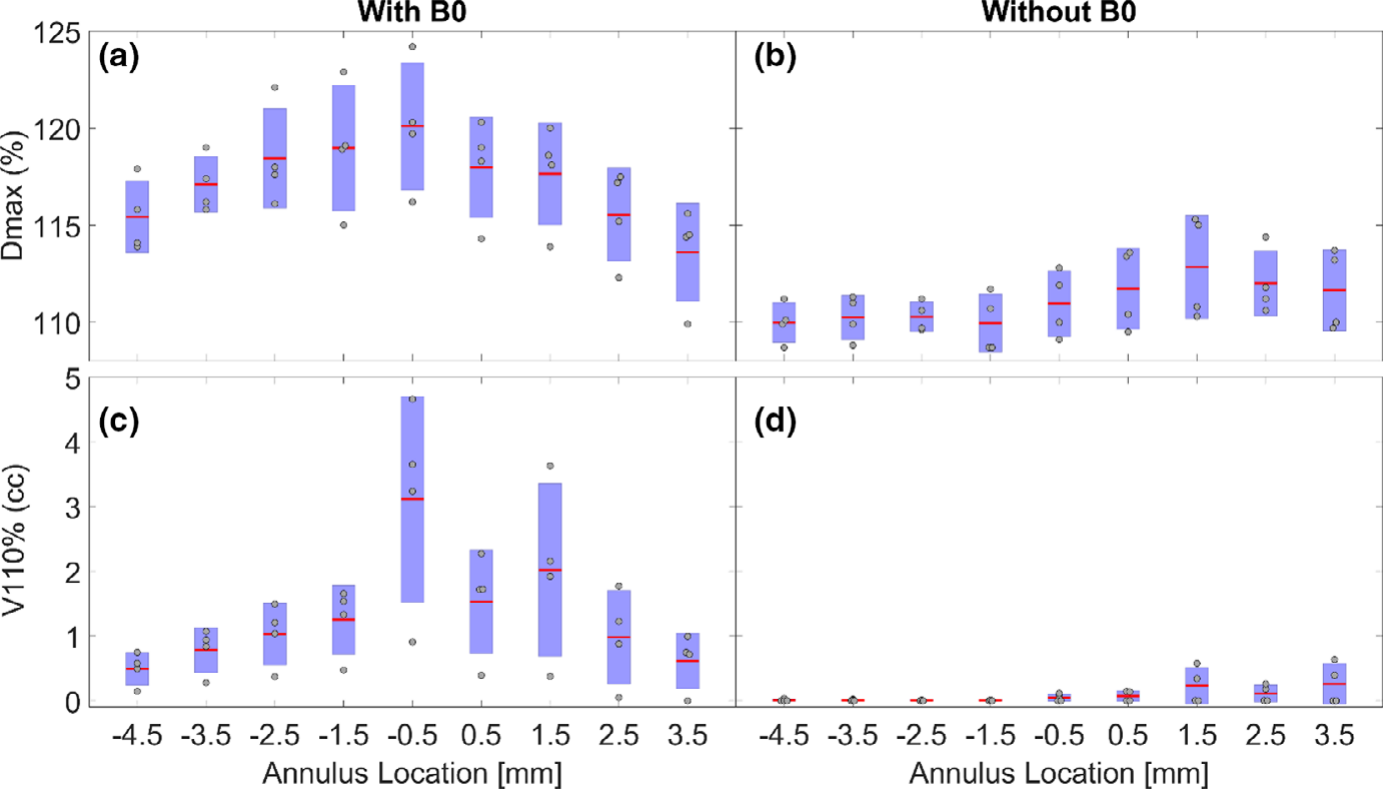
Location and volume of ERE hot spots for an air cavity size of 3 cm. Dmax (%) and V110% (cc) in the present of B field (a,c) and in absence of B field (b,d). Negative values on the x‐axis represent the annuli inside the cavity. Positive values on the x‐axis represent the annuli outside the cavity (in the tissue).

The location and volume of ERE hot spots were estimated by generating annulus rings of 1 mm as shown in Figs. 9(a)–9(d) The annuli rings inside the cavity (or the rectal wall) are represented on the negative x‐axis and the rings outside the cavity are represented on the positive x‐axis. As shown in Fig. 9(a), in the presence of magnetic field, hot spots higher than 115% are located 5 mm inside and 3 mm outside of rectum‐air interface. Higher hot spots (>117%) are located within the first 3 mm inside and 2 mm outside of the rectum‐air interface. Without magnetic field [Fig. 9(b)], hot spots are within 110 % inside and <113% outside the rectum‐air interface. In terms of volume receiving 110 % or higher of prescription dose, there is a significant increase in hot spots within the first 3 mm inside and 2 mm outside rectal interface with magnetic field [Fig. 9(c)], whereas without magnetic field there is a slight increase in hot spot volume outside of rectal interface (9d). Considering the first 3 mm inside the rectal interface, the maximum dose increased from 110.4 ± 0.5% without B field to 119.2 ± 0.8% with B field. Similarly, for the 2 mm outside the rectal interface, there was an increase in maximum dose from 112.3 ± 0.9% without B field to 117.8 ± 0.2% with B field.

## 4 DISCUSSION

In this study, we investigated the dosimetric effect of varying air cavity size on rectum plans for patients treated on a hybrid 1.5 T Unity MR‐linac system. We found that, in the absence of a magnetic field, the impact of varying air cavity size on plan dosimetry was limited to decreased attenuation due to increasing air volume and the variations seen were within statistical uncertainty. In the presence of a magnetic field, in addition to decreased attenuation due to increased air cavity, the curving path of electrons (or the electron return effect) at the tissue‐air interface affected the dosimetric parameters. In terms of location, most of the hot spots due to ERE were located at the posterior interfaces of rectum wall. This is because the target is more posteriorly located resulting in higher fluence contribution from the posterior beams than the anterior beams for both prone and supine positions. PTV coverage degraded as a function of air cavity in relation to tumor volume. Because the air cavity was mostly located in the high‐dose PTV volume, ERE effect on low‐dose PTV coverage was minimal. PTV 50 typically represents the rectal tumor while PTV45 represents the nodal volume. Air cavities drawn within PTV45 would have included bowel so we limited the air volumes within the high‐dose volume only. The polynomial fit to the PTV hot spot parameters enables predicting the hot spot values based on air cavity size during online plan adaptation for MR‐guided RT. Appropriate intervention can then be taken before the start of the treatment (asking the patient to relieve or ask the patient to get down etc.) or during the treatment because of appearing air volume.

The annulus calculation showed that hot spots and volumes increase with increasing air cavity diameter. The hot spot reaches its maximum value at the annulus diameter of ~3 mm which approximates the rectal mucosa and submucosa layer. Annulus calculation was done both inside and outside the rectal‐air interface as the electron density assignment from tissue to air within the calculation voxels may not be a step function but rather a gradual transition. In this study, a beam arrangement similar to that on a conventional linac was used. Future studies will investigate if alternate beam arrangement can further minimize the ERE due to the presence of air cavity. Since the air cavity can appear or disappear during treatment, our analysis helps in predicting the severity of hot spots without doing an exhaustive dose calculation.

**FIG. 10:**
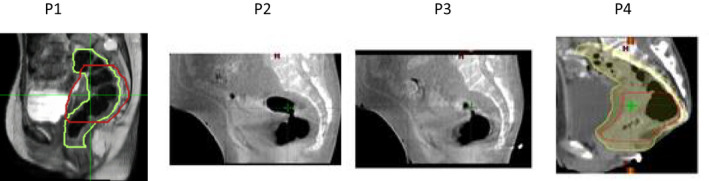
Clinical examples of typical air cavity size seen on the MR (P1) and CBCTs of four different patients.

In our clinical practice, based on the MR and CBCT scans, we have seen air cavities that are more often tubular rather than spherical (Fig. 10). The size of these cavities varies from patient to patient with pockets of air approaching average diameter up to 5 cm or even larger. Even if the entire rectum cavity does not contain the same air diameter, our results are still clinically relevant in determining the hot spots near the largest air cavity especially if the air pocket is located in the high‐dose PTV.

Finally, the results presented here assumed that the air cavity is present for the entire course of treatment which may not be the case. The dosimetric effect of air cavities is reported as a percent of the prescription or percent volume. Even though the results are shown for the entire course of treatment, they are more appropriate for individual dose fractions since the air cavity may not be present for the entire course. Our study helps put things in perspective for the planner regarding the magnitude and location of hot spots to expect on a given day depending on the volume of the air cavity.

## 5 CONCLUSIONS

In this study, we investigated the effect of electron return effect on rectum planning dosimetry for MR‐guided RT. Our work shows that the location and magnitude of hot spots are dependent on the size of the air cavity. We also showed that tumor coverage degrades as a function of air cavity in relation to tumor volume. The study has a potential to help physicists and physicians take appropriate intervention for daily adaptation based on the air cavity size for rectum patients treated on the Unity MR‐linac machine.

## CONFLICT OF INTEREST

None.
